# Prenatal maternal salivary hormones and timing of tooth eruption in early childhood: a prospective birth cohort study

**DOI:** 10.3389/froh.2025.1663817

**Published:** 2025-11-18

**Authors:** Ying Meng, Ruqian Yang, Nora Alomeir, Thomas G. O’Connor, Jerod M. Rasmussen, Felicitas B. Bidlack, Jin Xiao

**Affiliations:** 1 School of Nursing, University of Rochester, Rochester NY, United States; 2 Eastman Institute for Oral Health, University of Rochester Medical Center, Rochester NY, United States; 3 Departments of Psychiatry, Neuroscience, Obstetrics and Gynecology, University of Rochester, Rochester, NY, United States; 4 Department of Pediatrics, University of California, Irvine, CA, United States; 5 Craniofacial Biology and Bioengineering, ADA Forsyth Institute, Somerville, MA, United States

**Keywords:** tooth eruption, prenatal stress, cortisol, sex steroid hormones, thyroid hormones

## Abstract

**Background:**

Although the mechanisms underlying tooth eruption are not fully understood, the prenatal maternal milieu, particularly stress exposures, appears to play an important role in dental development. Yet, limited research has investigated the influence of prenatal stress and stress-related hormones on tooth eruption.

**Methods:**

This study included 142 mother-child dyads from a birth cohort to examine associations between prenatal stress, stress-related hormones, and primary tooth eruption. The number of erupted teeth was assessed by dentists at child visits through 24 months of age. Maternal prenatal depression and anxiety diagnoses were extracted from medical records as a proxy for stress. Stress-related hormone concentrations, including cortisol, estradiol, progesterone, testosterone, triiodothyronine (T3), and thyroxine (T4), were measured from salivary samples collected in late pregnancy. Generalized linear models were used to assess associations between prenatal stress, stress-related hormones, and tooth eruption, adjusting for relevant covariates.

**Results:**

Eruption timing varied within our cohort: 15.2% of children had at least one erupted tooth by 6 months, and 25% had all 20 primary teeth by 24 months. Correlations in tooth counts across visits ranged from 0.15 to 0.57. Several prenatal maternal hormones, including cortisol, estradiol, progesterone, testosterone, and T3, were significantly and positively associated with the number of erupted teeth at individual visits (*p* < 0.05). Particularly, higher prenatal cortisol levels were associated with more erupted teeth at 6 months, corresponding to an average difference of ∼4 teeth between the lowest and highest cortisol levels.

**Conclusion:**

Maternal salivary hormone levels in late pregnancy may contribute to variations in primary tooth eruption during the first two years of life.

## Introduction

The timing of tooth eruption is critical for oral health. Tooth eruption varies widely (1–3), with most children exhibiting between 2 and 11 erupted teeth by 12 months of age (2). Early and delayed eruption can disrupt dental alignment, contribute to malocclusion (4–6), compromise enamel quality, and increase the risk of dental caries (3, 7). Beyond oral health, early tooth eruption has also been proposed as a biomarker for accelerated biological aging in children (8).

Despite its clinical importance, the biological mechanisms regulating tooth eruption remain incompletely understood, though both genetic and environmental factors play critical roles (9, 10). Primary tooth development begins *in utero* (11, 12), making pregnancy a sensitive window when maternal and environmental exposures may influence dental outcomes. Previous studies have linked deviations in eruption timing to prenatal maternal smoking, suboptimal nutrition, and socioeconomic adversity (3, 13–15). Socioeconomic adversity is closely tied to chronic stress (16, 17), and prenatal stress exposure has also been associated with other dental outcomes, including enamel defects and dental caries (18, 19). However, few studies have directly examined whether prenatal stress specifically modulates the timing of tooth eruption.

This study aimed to address this research gap. Mechanistically, stress activates the hypothalamic-pituitar*y* axis and alters concentrations of cortisol (20, 21) as well as other hormones, such as sex steroids (22) and thyroid hormones (23). These hormones are involved in bone development and the metabolism of vitamin D and calcium (24–28), both of which are implicated in dental development and tooth eruption (29–32). This prior evidence suggests a plausible pathway linking stress-related hormones to dental development. Accordingly, in this study we evaluated both maternal prenatal stress and stress-related hormone levels in relation to tooth eruption.

## Method

### Study design and cohort

The current study uses data from a prospective birth cohort that recruited 186 socioeconomically disadvantaged pregnant women during late pregnancy and subsequently enrolled their newborn children between 8/2017 and 11/2022 (33). The inclusion criteria for the current study were: 1) mothers provided saliva samples during the late second or third trimester, and 2) tooth eruption information was available for children at any of the 6-, 12-, 18-, or 24-month study visits. The final analytic sample included 142 mother-child dyads. The birth cohort study was approved by the university's Research Subject Review Board (#1248). Written informed consent was obtained from all participants, and consent for minors was obtained from their legal guardians.

### Tracking tooth eruption

Comprehensive oral examinations were performed on children at multiple visits (1 week, 2, 4, 6, 12, 18, & 24 months) up to 24 months of age by one of three calibrated dentists using standard equipment. A tooth was considered erupted if its cusp was visible above the gum line. The number and type of erupted teeth were recorded at each visit for each child.

### Prenatal stress and hormone measures

Prenatal stress was not directly measured in our participants; instead, a proxy measure was used, based on a documented diagnosis of depression or anxiety during pregnancy in the medical record, according to previous studies (34, 35). The variable was coded as the presence or absence of depression or anxiety. Saliva samples were collected once from each woman during late pregnancy. A detailed description of salivary hormone assessment has been provided in our previous publication (36). Briefly, prior to sample collection, participants were instructed to refrain from eating, drinking, or brushing their teeth for 2 h. Each woman provided approximately 3–5 ml of unstimulated saliva during the late 2nd or 3rd trimester by spitting into a sterile centrifuge tube. Salivary hormone levels of cortisol, estradiol, progesterone, testosterone, triiodothyronine (T3), and thyroxine (T4) were measured using the MILLIPLEX MAP Multi-Species Hormone Magnetic Bead Panel (Cat# MSHMAG-21K, Merck Millipore, Darmstadt, Germany). Results were obtained on a Luminex 200 instrument (Luminex, USA) and reported based on standard curve values.

### Covariates

Covariates included in the model were selected based on previous literature (3, 15, 37). Maternal and child demographic and socioeconomic information (maternal age, educational level, marital status, employment status, parity, smoking status, child sex, child race/ethnicity, birthweight, and breastfeeding status) was obtained through self-reported questionnaires and medical records. Maternal medical conditions (diabetes and hypertension) were self-reported and confirmed using electronic health records. Gestational age at sample collection was calculated from the due date and the sample collection date. Breastfeeding status was determined primarily from the 6-month visit. If data at the 6-month visit were missing, information from earlier visits (2- and 4-month) was used; if unavailable, breastfeeding data from the 12-month visit were used.

### Statistical analysis

All hormone levels were natural log-transformed to approximate a normal distribution. To reduce model complexity and avoid overfitting, covariates were selected for the final models through univariate analyses between each covariate and the number of erupted teeth at each visit, using a *p*-value threshold of 0.2. The final models included maternal age, educational level, employment status, nulliparity, smoking status, diabetes, hypertension, and child sex, race/ethnicity, birthweight, and breastfeeding status. Pearson and Spearman correlations were used to assess relationships among stress-related hormones and, separately, among the number of erupted teeth across visits. Group-based trajectory modeling was applied to identify distinct patterns of tooth eruption over time.

Because the outcome variable, the number of erupted teeth, was a count variable, generalized linear models were used to examine associations between prenatal stress, stress-related hormones, and tooth eruption at each visit, adjusting for covariates. A Poisson distribution with a log link function was used for the 12-, 18-, and 24-month visits, while a negative binomial distribution was applied at the 6-month visit to account for overdispersion. Overall associations across all visits were assessed using negative binomial mixed-effects models. Sensitivity analyses excluded infants born with low birthweight (<2,500 grams; *n* = 1), and cases with maternal hormone levels identified as outliers, defined as values beyond the median ± 4 × interquartile range. All statistical analyses were conducted using STATA 18.0 (College Station, TX, USA).

## Results

### Characteristics of participants

The characteristics of the mother-child dyads (*n* = 142) are summarized in Table 1. Most mothers were employed (>53%), had a high school education or lower (60%), had more than one pregnancy (>76%), and did not breastfeed around 6 months postpartum (59%). All children were born full-term, with an average birthweight of 3,308 grams. More than half of the children were African Americans (>52%).

**Table 1:** Characteristics of the mother-child dyads included in the current study (*n* = 142).

Variable	*N* (Percentage)	Mean (SD)
Maternal characteristics		
Age (years)		27.6 (5.4)
Smoking	21 (14.8%)	
Employed	76 (53.5%)	
Education:		
High school or lower	85 (59.9%)	
Associate degree	21 (14.8%)	
College or higher	36 (25.4%)	
Married	30 (21.1%)	
Nulliparous	47 (33.1%)	
Prenatal diabetes	9 (6.3%)	
Prenatal hypertension	19 (13.4%)	
Gestational weeks at sample collection		33.2 (3.4)
Breastfeeding-6 months	58 (40.9%)	
Child characteristics		
Sex: Female	75 (52.8%)	
Race:		
White	27 (19.0%)	
Black	74 (52.1%)	
Hispanic	20 (14.1%)	
Others	21 (14.8%)	
Birthweight (g)		3,308.5 (434.2)

**Figure 1:**
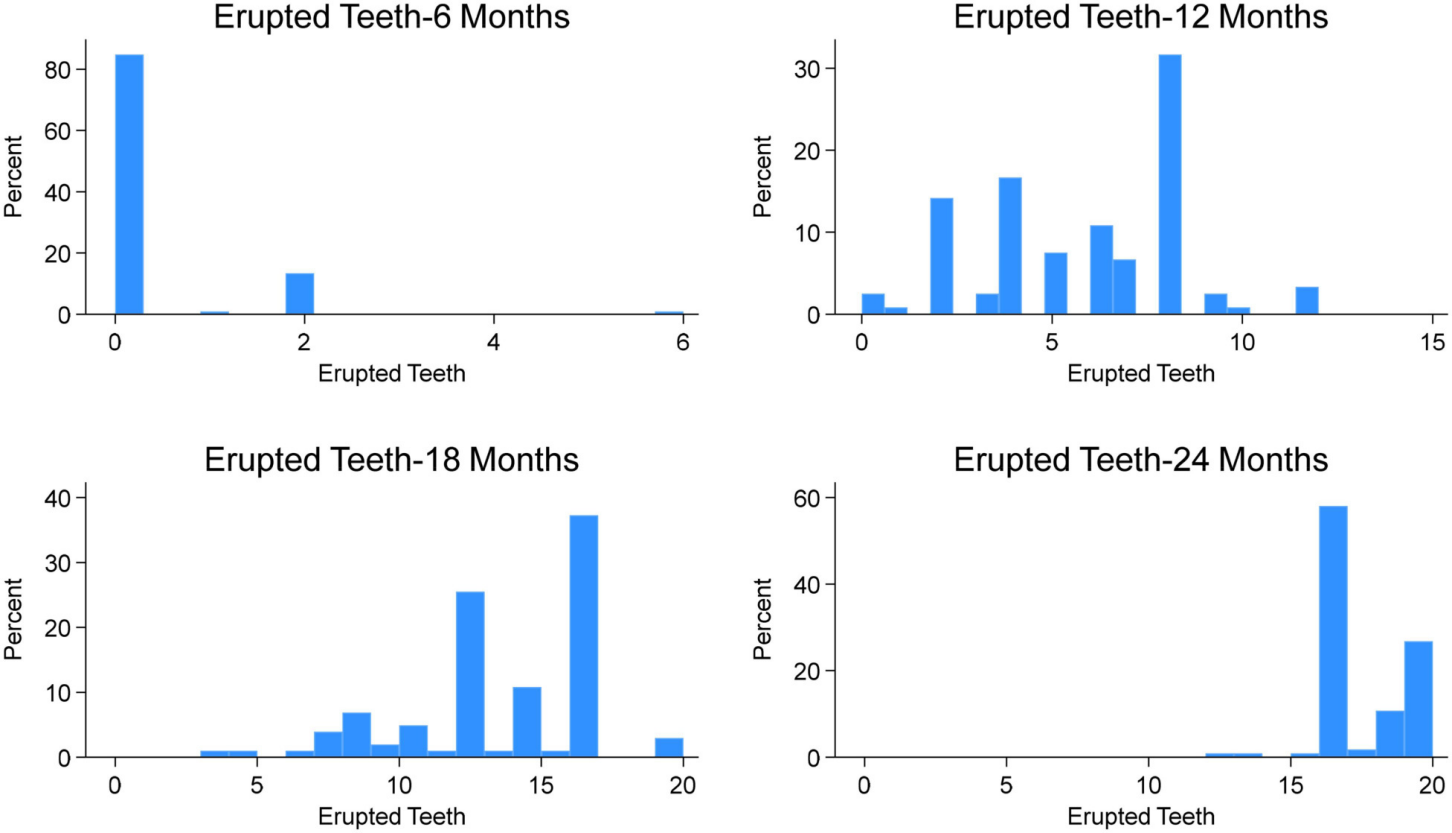
Erupted teeth at different visits from 6 to 24 months.

**Figure 2:**
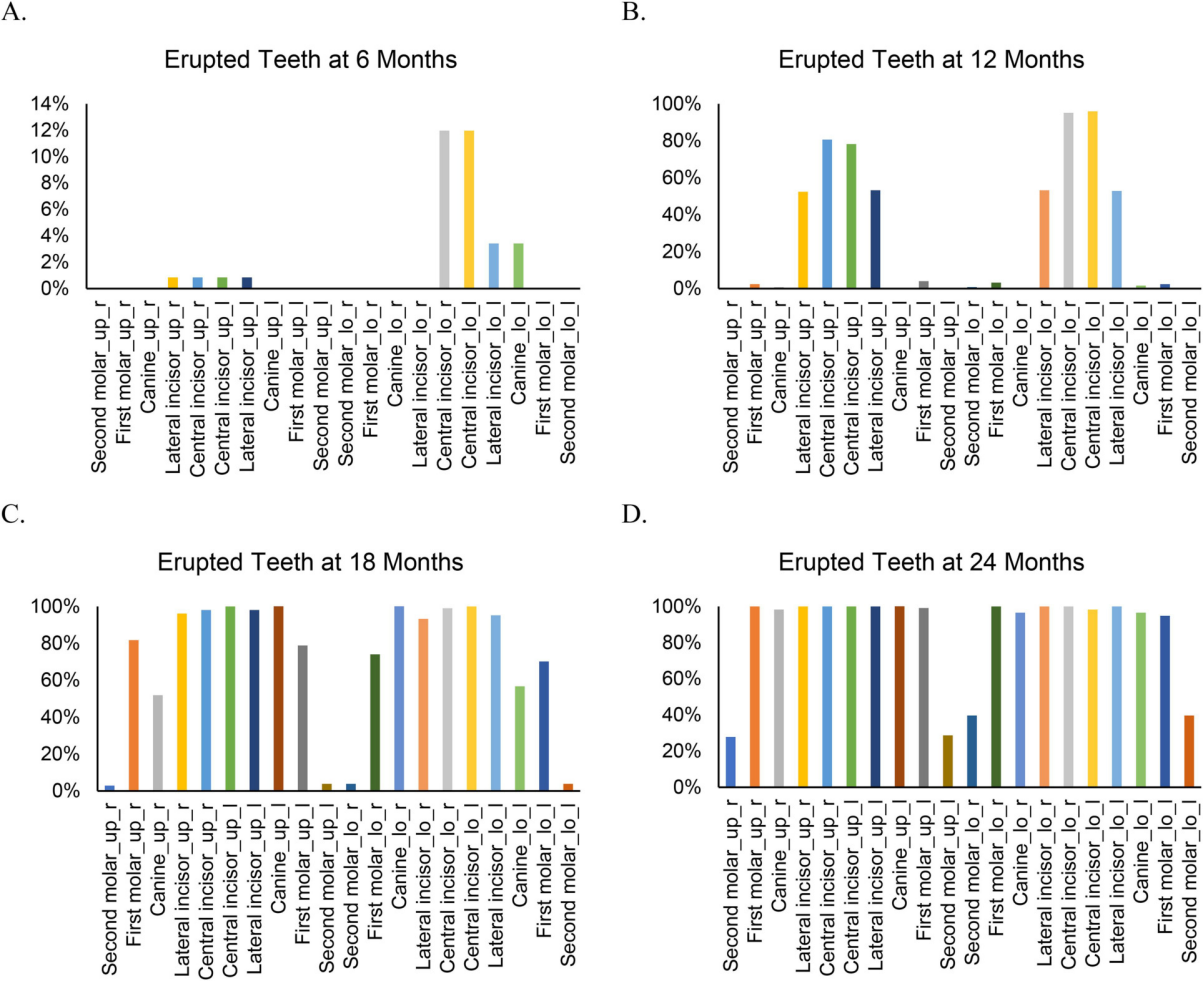
Type of erupted teeth at different visits from 6 to 24 months. “up” indicates upper and “lo” indicates lower. “r” indicates right and “l” indicates left.

By 6 months of age (Figure 1), 17 children (15.2%) had erupted teeth, ranging from 1 to 6 (mean = 0.33, SD = 0.87). The lower central incisors (12%) were the most common teeth erupted at this age (Figure 2A). By 12 months, 3 children (2.5%) had no erupted teeth, while the median number of erupted teeth was 6 (range: 0–12; mean = 5.76, SD = 2.69). More than 95% of children had erupted lower central incisors, and approximately 80% had erupted upper central incisors (Figure 2B). At 18 months, all children attending the study visit (*n* = 102) had erupted teeth, with a median of 14 (range: 3–20; mean = 13.13, SD = 3.39). By 24 months, the median number of erupted teeth was 16 (mean = 17.18, SD = 1.85), and 25% of children had a full set of 20 primary teeth (Figure 1). Fewer than 40% had erupted upper and lower second molars (Figure 2D).

**Figure 3:**
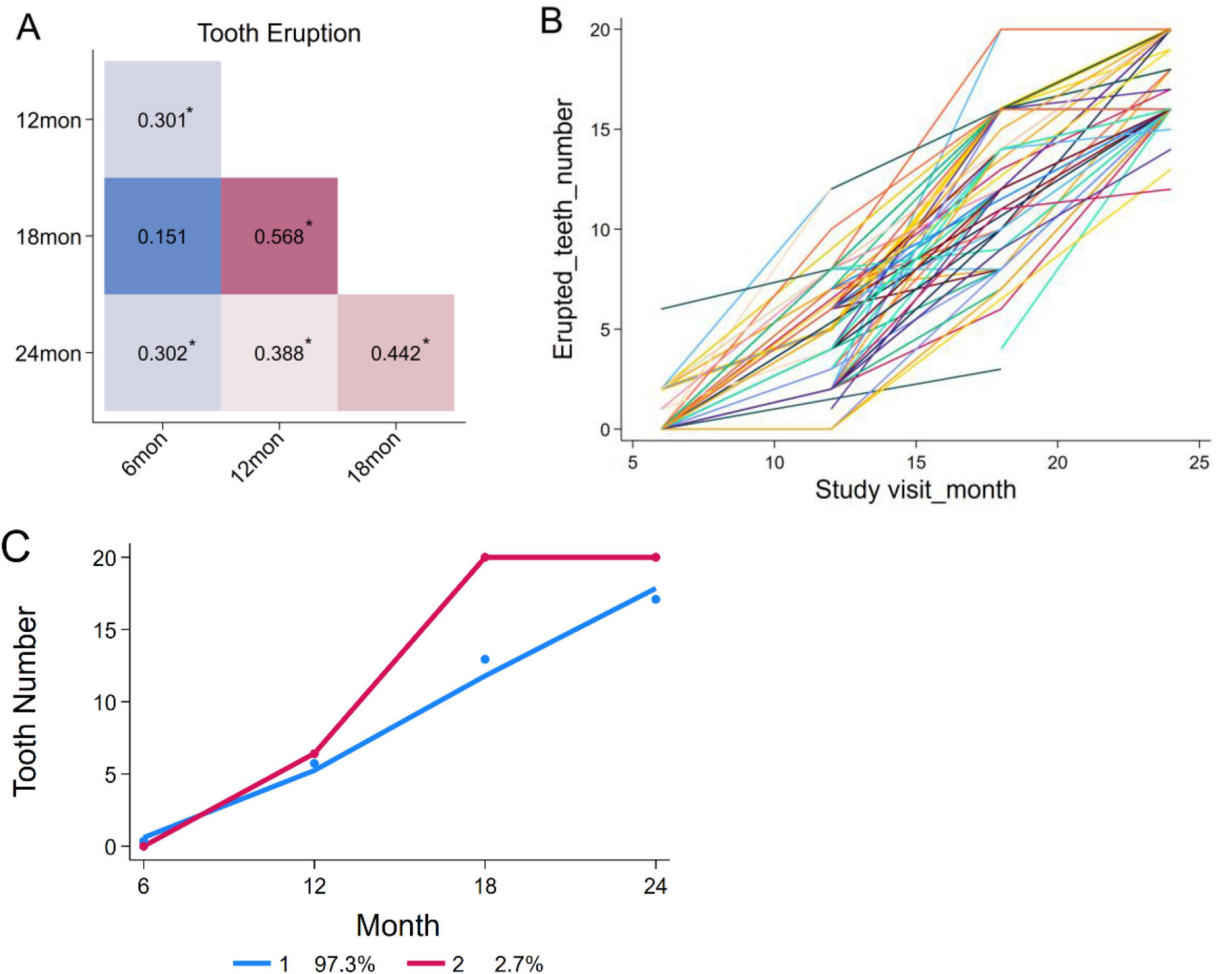
Correlations among the number of erupted teeth at different visits and individual growth trends. **(A)** Correlations among tooth counts from 6 to 24 months of age. **(B)** Variation in tooth eruption patterns among individual children. **(C)** Two distinct tooth eruption patterns identified by group-based trajectory modeling.

Spearman's rank-order correlation was used to assess associations among tooth counts across visits. Significant correlations were observed (*p* < 0.01) for all pairs except between 6 and 18 months. Significant correlation coefficients were weak to modest, ranging from 0.30 to 0.57 (Figure 3A). However, the intraclass correlation coefficient (ICC) across visits was close to “0” (ICC = 7.24 × 10^−10^), suggesting limited stability in individual eruption patterns (Figure 3B). Group-based trajectory modeling identified two distinct tooth eruption patterns during the first two years of life, based on the lowest Bayesian information criterion (BIC, Figure 3C): a continuous growth pattern (97.3% of children) and a rapid growth pattern between 12 and 18 months (2.7% of children).

**Figure 4:**
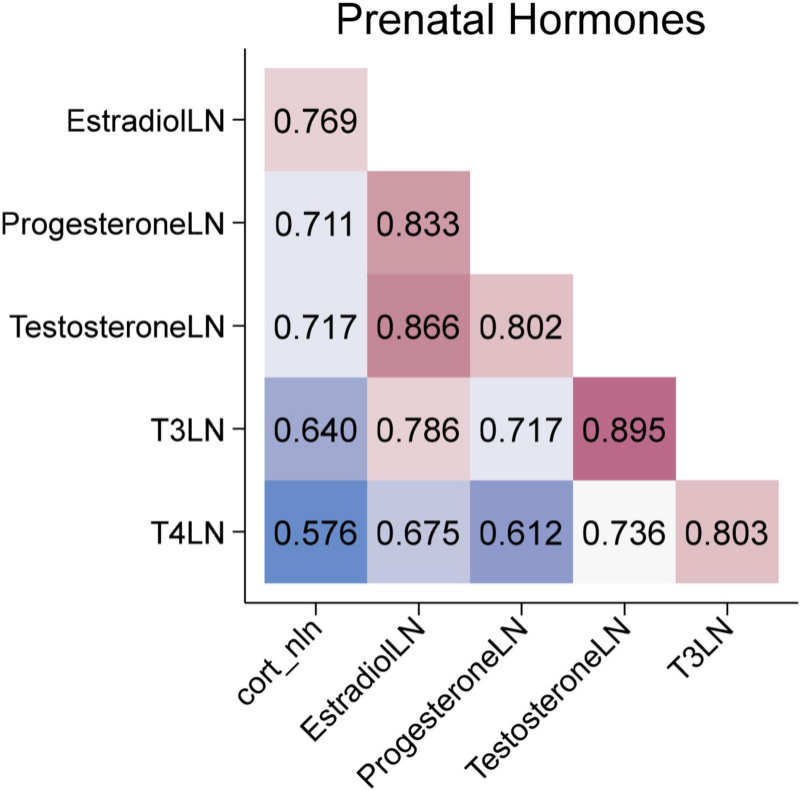
Correlations among maternal prenatal hormones.

During pregnancy, 36.6% of women had a documented diagnosis of depression or anxiety. Levels of stress-related hormones measured in maternal salivary samples collected during the late 2nd or 3rd trimester are presented in Table 2. All hormones were significantly correlated (*p* < 0.001), with correlation coefficients ranging from 0.58 to 0.89 (Figure 4). No significant associations were observed between depression or anxiety diagnoses and hormone levels.

**Table 2:** The hormone levels in saliva samples included in the current study (*n* = 142).

Prenatal hormone (pg/ml)	Median	IQR
Cortisol	5,840	6,420
Estradiol	595	890
Progesterone	10,010	16,910
Testosterone	620	1,550
T3	340	1,480
T4	80	940

### The relationship between prenatal depression/anxiety, salivary hormones, and tooth eruption

Due to the small-to-moderate correlations and low ICC for the number of erupted teeth across visits, associations between prenatal depression/anxiety, salivary hormones, and tooth eruption were first assessed separately at each child visit. Prenatal maternal depression/anxiety was not significantly associated with the number of erupted teeth at any time point (*p* > 0.05). In contrast, several prenatal stress-related hormones, including cortisol, estradiol, progesterone, testosterone, and T3, were positively associated with tooth counts (Table 3).

**Table 3:** The associations between prenatal maternal hormones and tooth eruption longitudinally and cross-sectionally.

Prenatal hormone	Tooth number at 6 months		Tooth number at 12 months		Tooth number at 18 months		Tooth number at 24 months		Tooth number across all visits^a^	
	*β*	95%CI	*β*	95%CI	*β*	95%CI	*β*	95%CI	*β*	95%CI
Cortisol	**0**.786*	**0.120, 1.452**	0.076	−0.019, 0.171	0.023	−0.037, 0.084	0.020	−0.004, 0.043	0.025	−0.028, 0.077
Estradiol	0.353	−0.090, 0.796	**0**.078*	**0.002, 0.154**	0.035	−0.011, 0.081	0.018	−0.001, 0.037	0.026	−0.013, 0.065
Progesterone	0.326	−0.159, 0.812	0.057	−0.008, 0.123	0.004	−0.030, 0.037	**0**.018*	**0.003, 0.034**	0.016	−0.016, 0.049
Testosterone	0.008	−0.216, 0.232	**0**.044*	**0.004, 0.084**	0.022	−0.002, 0.047	**0**.010*	**0.001, 0.019**	0.013	−0.008, 0.033
T3	0.030	−0.199, 0.258	0.023	−0.014, 0.061	**0**.026*	**0.002, 0.050**	0.012**	**0.003, 0.020**	0.012	−0.007, 0.031
T4	0.158	−0.106, 0.421	0.020	−0.028, 0.068	0.016	−0.014, 0.046	0.011	−0.0003, 0.022	0.007	−0.017, 0.032

GLM model adjusted for infant sex, race/ethnicity, infant birthweight, breastfeeding prior to 6 months, prenatal maternal diabetes, hypertension, smoking, age, education, employment, and nulliparity. All prenatal hormones were natural log transformed.

Bold values are significant results.

a Estimations were derived from negative binomial mixed-effects models.

* *p* < 0.05. **p < 0.01.

Specifically, higher prenatal cortisol levels were associated with a greater number of erupted teeth at 6 months (*β* = 0.79, 95% CI: 0.12–1.45), corresponding to an average difference of ∼4 teeth between the lowest and highest cortisol levels. At 12 months, higher estradiol (*β* = 0.08, 95% CI: 0.002–0.15) and testosterone (*β* = 0.04, 95% CI: 0.004–0.08) were positively associated with tooth count, corresponding to average differences of 0.4 and 0.3 teeth, respectively. At 24 months, progesterone (*β* = 0.02, 95% CI: 0.003–0.03) and testosterone (*β* = 0.01, 95% CI: 0.001–0.02) were associated with tooth count, corresponding to average differences of 0.1 and 0.07 teeth. T3 levels were also positively associated with tooth counts at 18 months (*β* = 0.03, 95% CI: 0.002–0.05) and 24 months (*β* = 0.01, 95% CI: 0.003–0.02), corresponding to average differences of 0.2 and 0.09 teeth, respectively, between the lowest and highest hormone levels.

When overall associations across all visits were assessed, the results were non-significant, likely reflecting the low ICC and weak correlations across visits. Most findings remained robust in sensitivity analyses; however, associations between cortisol and tooth count at 24 months (*β* = 0.03, 95% CI: 0.0002–0.05) and between estradiol and tooth count at 24 months (*β* = 0.02, 95% CI: 0.0005–0.04) became significant. In contrast, associations with estradiol (*p* = 0.09) and testosterone (*p* = 0.05) at 12 months were attenuated.

## Discussion

Overall, our findings indicate that correlations in the number of erupted teeth across visits during the first two years of life were weak to modest, suggesting variability in dental growth patterns among individual children. Several prenatal salivary hormones were positively associated with the number of primary teeth at specific time points, with more associations observed at 24 months. However, the strongest associations emerged earlier, particularly between prenatal cortisol levels and tooth count at 6 months. These findings may suggest potential biological mechanisms through which prenatal stress may influence the timing of tooth eruption.

In our cohort, the sequence of primary tooth eruption aligned with prior studies (38), yet children exhibited relatively delayed tooth eruption by 12 months and more advanced development between 12 and 24 months compared to previous studies (3, 38). Such differences may be explained by variability in race/ethnicity and socioeconomic status across study populations. Interestingly, we observed heterogeneous tooth eruption patterns, with some children experiencing rapid tooth emergence in infancy and others showing accelerated eruption during toddlerhood. Two primary eruption trajectories were identified using group-based trajectory modeling. However, the weak to modest correlations across visits suggest that additional, unidentified eruption trajectories may exist. Future studies with larger, more diverse samples are needed to characterize these potential subgroups and to assess predictors of interindividual variation in eruption timing.

To date, evidence linking prenatal hormone levels with tooth eruption timing in offspring remains sparse. Vucic and colleagues found no significant associations between maternal thyroid hormones and dental development at age 9 (31), consistent with our null findings for T4. However, we observed significant associations between prenatal maternal T3 levels in late pregnancy and the number of erupted teeth at 18 and 24 months. Because T3 was not measured in the prior study (31), our findings may highlight a novel association. Biologically, T3 plays a critical role in bone development (26) and mineral homeostasis (39, 40), which may similarly influence odontogenesis and help explain its relationship with tooth eruption.

Given the relatively high correlations among the measured hormones, it remains unclear which ones play a key role in modifying eruption timing. Cortisol, estradiol, progesterone, testosterone, and T3 are all implicated in bone development and calcium/vitamin D metabolism (24–28). Among these, prenatal cortisol showed the strongest association with tooth count at 6 months. Biologically, cortisol has been shown to impair osteoclastogenesis and osteoblastogenesis and promote apoptosis of osteoblasts and osteocytes (41), although its role in odontogenesis remains unclear. Cortisol also affects vitamin D and calcium availability through malabsorption, hypercalciuria, and bone resorption (24, 42, 43). Experimental studies have demonstrated that calcium and vitamin D supplementation during pregnancy accelerates tooth eruption in mice (44), yet evidence in humans remains inconsistent (15, 45, 46). Our observation that high prenatal cortisol was linked to earlier tooth eruption may also parallel findings that prenatal stress accelerates biological aging processes such as epigenetic modifications and telomere shortening (47–49).

Although associations between sex steroid hormones (estradiol, progesterone, and testosterone) and tooth eruption were statistically significant, effect sizes were small, suggesting limited clinical impact. These hormones are essential for fetal development and birthweight, both linked to eruption timing (9, 15, 50). Sex steroid hormone levels may also indirectly reflect vitamin D sufficiency, which regulates placental hormone production (27). Estradiol additionally modulates osteoblasts partly through the RANKL-OPG-RANK pathway (28), which may also influence odontogenesis. Nevertheless, given the small magnitude of effects observed, these associations should be interpreted cautiously.

Several limitations in this study warrant consideration. First, the ICC for erupted tooth counts across visits was close to zero, indicating low stability of eruption over time within individuals. In practical terms, having more teeth in early infancy did not reliably predict having more teeth later. Therefore, interpretations of mixed-effects and cross-sectional models should be made with caution. Second, prenatal stress was assessed during maternal diagnoses of depression or anxiety, which likely underestimated stress exposure, particularly in a cohort characterized by socioeconomic adversity. While salivary cortisol provided an objective biomarker of stress (51), future studies should incorporate validated stress questionnaires, such as perceived stress scale and stressful life events, alongside biomarkers to capture stress more comprehensively. Third, hormone levels were measured from a single salivary sample. This approach does not account for diurnal cortisol fluctuations, which better reflect overall activity of the hypothalamic-pituitary-adrenal axis (52). Therefore, future studies are recommended to collect multiple saliva samples to capture cortisol diurnal variation. For the other hormones, prior studies indicate that salivary and blood concentrations show moderate to strong correlations (53, 54). suggesting that salivary hormone levels may reflect systemic levels. Finally, maternal biospecimens were collected in late pregnancy, a period corresponding to crown formation. However, primary tooth development begins as early as ∼6 weeks of gestation (55), and the influence of hormones may vary by gestational stage. Future studies should consider assessing hormone levels at multiple time points throughout pregnancy to better examine potential time-specific effects on tooth development.

Despite these limitations, this study has several strengths. As a prospective cohort, maternal and child data were collected longitudinally, reducing recall bias. Dental assessments were performed by calibrated dentists using standardized protocols, ensuring data quality and consistency. Moreover, we examined a broad panel of hormones related to stress and fetal development, enabling exploratory analyses of potential biological mechanisms linking prenatal stress with primary tooth eruption.

## Conclusion

Maternal salivary hormone levels, particularly cortisol, measured in late pregnancy were potentially associated with tooth eruption in children during the first two years of life. Larger, more diverse cohorts with repeated biomarker assessments are needed to clarify the biological mechanisms linking prenatal stress to tooth eruption.

## Data Availability

The original contributions presented in the study are included in the article/Supplementary Material, further inquiries can be directed to the corresponding author.
