# Supplementary material for: Prenatal maternal salivary hormones and timing of tooth eruption in early childhood: a prospective birth cohort study
Source: Front Oral Health. 2025 Nov 18;6:1663817. doi: 10.3389/froh.2025.1663817 (PMC12669127; doi:10.3389/froh.2025.1663817)
Supplement: Supplementary file 1 [file Supplementaryfile1.docx]

**APPENDIX B: Questionnaire for Mother**

| **Please read each question carefully. Fill in the blank or place a check mark (*✓ )* in the BOX next to the answer that best describes you**. |
| --- |

***Part 1: Social-demographic background***

1. What is your current work status?

_1_ I am currently employed

_2_ I am currently unemployed

2. If currently employed, what is your most recent occupation?

________________________________________________

3. What is the highest grade you completed?

_1_ Middle school

_2_ High school

_3_ More than High School

_4_ College level

_5_ Postgraduate level

4. What is your marital status?

_1_ Married

_2_ Single

_3_ Separated

_4_ Divorced

_5_ Widowed

_6_ Other

***Part 2: History of yeast infection***

5. Do you have history of yeast infection?

_1_ No

_2_ Athlete’s foot

_3_ Ringworm

_4_  Cradle cap (when you were a child)

_5_ Oral thrush

_6_ Denture-related stomatitis

_7_ Angular stomatitis

_8_ Systemic candidiasis

_9_ other: ______________

6. Have you had long term (>3month) antibiotics use?

_1_ No

_2_ Yes, please specify ____________________

*7.* Have you had antifungal therapy (treating yeast infection) in the past 3 month?

_1_ No

_2_ Yes, please specify ___________________

***Part 3: Hygiene***

*8.* How often do you brush teeth?

_1_ Twice/daily

_2_ Once/daily

_3_ Not everyday

_4_ Never

***Part 4: Oral status of other children***

9. How many children you have? ____________

**If you don’t have any other children, please skip question 10 and 11.**

10. In the last 12 months did any one of your other children have a toothache or injury that needed care right away in emergency room or dental urgent care?

_0_ Yes

_1_ No

11. When was the last time your other child/children had a dental check-up?

_1_ In the past 12 months

_2_ 1 to 2 years ago

_3_ More than 2 years ago

_4_ Never

**THANK YOU FOR ANSWERING these QUESTIONS**

**Please return the completed questionnaire to the study staff**
